# North East England South Asia Mental health Alliance (NEESAMA): an exemplar of global north and global south collaboration to improve research, training and service delivery in mental healthcare

**DOI:** 10.1192/bji.2023.22

**Published:** 2023-11

**Authors:** Meetali Devgun, Caitlin Kittridge, Shekhar Seshadri, Jacqueline Rodgers, Aditya Narain Sharma

**Affiliations:** 1 Visiting Assistant Professor, School of Interwoven Arts and Sciences, Krea University, Bengaluru, India; 2 Psychology Placement Student, School of Psychology, Newcastle University, Newcastle upon Tyne, UK; 3 Emeritus Professor, SAMVAD (Support, Advocacy and Mental Health Interventions for Children in Vulnerable Circumstances and Distress), National Institute of Mental Health and Neuro Sciences (NIMHANS), Bengaluru, India; 4 Professor of Child Psychology, Population Health Sciences Institute, Newcastle University, Newcastle upon Tyne, UK; 5 Clinical Senior Lecturer and Honorary Consultant in Child and Adolescent Psychiatry, Translational and Clinical Research Institute, Newcastle University, Newcastle upon Tyne, UK. Email: aditya.sharma@ncl.ac.uk

**Keywords:** Global mental health, child and adolescent, adult, older people, collaboration

## Abstract

Despite the worldwide burden of mental illness and recent interest in global approaches to address this, progress on increasing awareness, lessening stigma, reducing the treatment gap, and improving research and training in mental health has been slow. In 2018, the North East England South Asia Mental health Alliance (NEESAMA) was developed as a collaboration between high-income (global north) and low- to middle-income (global south) countries to address this slow progress. This paper outlines how the joint priority areas for research, training and service delivery were identified across the life course (child and adolescent, adults and older people) between partner organisations spanning Afghanistan, Bangladesh, India, Nepal, Pakistan, Sri Lanka and the UK. It describes the progress to date and proposes a way forward for similar alliances to be forged.

## Background

Almost 1 billion people worldwide experience mental disorders, making global mental health an area receiving increasing attention. However, progress continues to be slow.^1^ The treatment gap between high-income countries and low- to middle-income countries (the global north and the global south) continues to be unacceptably wide, with 1 in 5 people in the global north receiving adequate treatment, in stark contrast to 1 in 27 people in the global south.^2^ The global south faces multiple challenges; mental healthcare often takes a back seat to physical healthcare and there is a high burden of mental illness due to the prevalence of social, emotional and physical deprivation.^3^ Stigma associated with mental illness may be a significant barrier to care, with cultural attitudes often equating mental illness with weakness or shame.^4^ Additionally, there is a shortage of mental healthcare providers and facilities and limited investment in mental health research, which results in significant unmet need.^5^ Addressing mental health needs in the global south is critical to improving overall well-being and reducing the burden of mental illness. This requires concerted efforts to tackle stigma and discrimination, improve access to mental healthcare and increase the availability of resources. Addressing these challenges requires investment in services, research and infrastructure, and a shift in cultural attitudes towards mental illness.

While the global south has the challenges outlined above in mental health service delivery systems and structures, the global north has a rapidly growing multi-ethnic society, which can pose unique issues. According to the 2019 population survey in England and Wales, the Asian and British Asian communities accounted for 8% of the total population, followed by other ethnic groups.^6^ Studies have shown low mental health service utilisation by ethnic minority groups, perhaps owing to a lack of culturally informed mental health assessment and intervention.^7^ By knowledge exchange with the global south, the global north can develop culturally informed interventions to enhance the accessibility, allocation and efficacy of mental health provision for ethnic minorities.

In 2018 the North East England South Asia Mental health Alliance (NEESAMA; www.neesama.org) was created in response to these challenges. NEESAMA is a collaboration between partners in South Asia, including Afghanistan, Bangladesh, India, Nepal, Pakistan and Sri Lanka, and England (Newcastle University and Cumbria, Northumberland, Tyne and Wear (CNTW) NHS Foundation Trust), with initial funding to support the development of the initiative provided by the British Council, CNTW NHS Foundation Trust and Newcastle University. NEESAMA aims to advance research, professional training and clinical service delivery in mental health, by establishing a partnership between policymakers, researchers and clinicians from the global north and the global south. It involves reciprocal and equitable knowledge exchange to support and facilitate improvement in mental health training and service delivery across and between all partner organisations.

## Priority areas, objectives and action plan

NEESAMA adopts a lifespan approach to mental health, incorporating objectives relating to children and adolescents, working age adults and older people. Across South Asia, a sizeable proportion of the population are children and adolescents, who frequently encounter restricted availability of mental health services, alongside increased exposure to adverse childhood experiences. Neurodevelopmental conditions and emotional and behavioural disorders comprise a considerable portion of the worldwide mental health burden for children and need to be prioritised in child mental health service development.^8^ NEESAMA recognises that cultural differences must be considered in relation to symptomatology, risk and protective factors, interventions and support for young people. NEESAMA aims to provide a vehicle for the development of evidence-based practices that are acceptable, valid, feasible and scalable for South Asia through the involvement and collaboration of representatives from each country, who, working together, can identify potential routes to vulnerability for children and young people, develop appropriate and validated identification and outcome measures, and evaluate promising practices in addressing the impact of adversity.

In relation to adult mental health, challenges such as low recruitment in research studies, which affects statistical power and effect sizes, paucity of validated assessment techniques and scalable interventions were identified as priority areas. An action plan was devised to address these concerns and it includes objectives related to supporting researchers to work with policymakers and other stakeholders to ensure scalability and sustainability beyond research studies. The plan also includes objectives related to conducting systematic reviews to identify appropriate screening instruments to estimate the epidemiology of serious mental illnesses and examine biopsychosocial factors in their development, to inform the development of culturally and ecologically valid interventions.

Lastly, given the increasing population of older people in the global south, coupled with a lack of experienced researchers in geriatric mental health and limited clinical training, NEESAMA aims to establish a clinical academic network focused on caring for older adults. The network will collect pilot data in India and Nepal on the provision of financial support and healthcare for this population, review the data, modify and potentially expand the data collection fields, and extend the survey to other countries.

Further, clinical and research training and service development were identified as common threads across the lifespan. The lack of mental health and allied professionals in the global south has been identified as a significant barrier to addressing the mental health needs of citizens, particularly children, young people and older adults.^9^ In light of such challenges, NEESAMA is committed to the provision of continuing professional development (CPD) training opportunities at a discounted cost in NEESAMA countries and identification and provision of support to early career researchers (ECRs) from each country to address the research gaps.

**Fig. 1:**
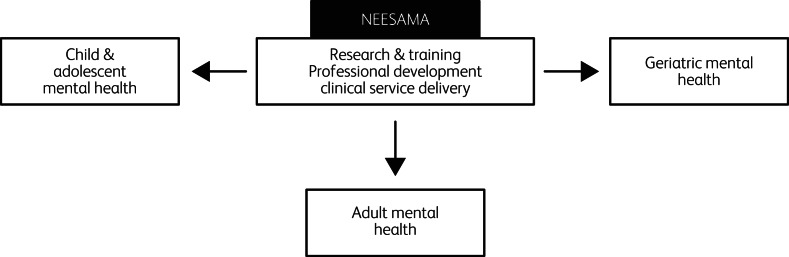
The North East England South Asia Mental health Alliance (NEESAMA) model.

The key priority areas identified by NEESAMA are aligned with the priorities in the World Health Organization's Mental Health Action Plan.^10^ The priority areas include (a) the identification of critical research questions focused on mental, neurodevelopmental and neurodegenerative disorders; (b) the identification of mechanisms for the enhancement of skill sets of clinicians working with children and adolescents, working-age adults and older adults (including the development of community-based services to cater to the needs of these age groups) and (c) ensuring the sustainability, longevity and impact of NEESAMA through the involvement of key policymakers and development of a clear and achievable action plan. Fig. 1 illustrates the multidimensional NEESAMA model (for more details, refer to www.neesama.org).

## Partnership effects

Since its inception in 2018, NEESAMA has held annual meetings (face to face and online) ensuring continued knowledge exchange between partner countries to enhance the progress of the goals and objectives of the alliance. During the last face-to-face annual NEESAMA meeting in 2019 in Bangladesh, the member countries organised a large-scale CPD panel discussion on suicide, dementia and depression across the lifespan, people in disadvantaged situations, and substance misuse at the Bangabandhu Sheikh Mujib Medical University (BSMMU) in Bangladesh. The CPD event was attended by several hundred mental health professionals in training at BSMMU. Several research projects have been conceptualised during the annual meetings and carried out in the following years. The collaboration between the partner countries has resulted in publications on geriatric mental health^11^ and mental health in the context of the COVID-19 pandemic^12^ in international peer-reviewed journals. Additionally, rapid reviews on childhood maltreatment and mood disorders and the psychological impact of COVID-19 on child and adolescent mental health, and a scoping review on psychotropic fixed-dose combination in India, are underway. The partnership has also resulted in the adaptation of the Anxiety Scale for Children – Autism Spectrum Disorder (ASC-ASD)^13^ in five South-Asian languages as well as the development and evaluation of the feasibility of a novel parent-mediated group intervention to address anxiety experienced by autistic children (Helping Your Anxious Child), which is being delivered in Sri Lanka and Bangladesh. NEESAMA also awarded ECR status, following a competitive process, to eight young professionals from the partner countries to ensure the continued growth and success of this scientific enterprise.

Further, NEESAMA has recently launched the NEESAMA Training and Research Academy (NEESAMA TARA), which aims to enhance the skill set of NEESAMA colleagues via a series of free, monthly online training sessions and webinars focusing on mental health that will be shared internationally through the alliance. TARA supports the ongoing objectives of NEESAMA to advance research, training and clinical service delivery within mental health internationally. Planned training events will address depression (treatment and related factors), mental health in Afghanistan, interpersonal therapy, qualitative research methods in mental health, cognitive impairment and dementia in Parkinson's disease, task shifting for maternal mental health in low-resource settings and biomarker research in mood disorders and non-invasive brain stimulation.

## Conclusions

The importance of international mental health alliances such as NEESAMA cannot be overstated. Mental disorders are a significant public health issue globally, wherein the global south contends with the problem of inadequate resources and services for mental healthcare and the global north grapples with an increasing ethnically diverse population and a lack of culturally sensitive mental healthcare provision. Thus, collaborations such as NEESAMA can foster a deeper understanding of cultural nuances affecting mental health and improve the development and delivery of culturally informed evidence-based interventions for mental illness in all the partner countries. Such alliances provide a platform for sharing knowledge, expertise and resources between partner countries to improve the quality of mental health research, education and clinical services.

Alliances between the global north and the global south can have far-reaching benefits in both settings, enabling the exchange of best practices, enhancing capacity building and resource sharing, and fostering the development of sustainable and locally relevant solutions to improve mental health services. By working together, countries can develop and implement policies and interventions that are culturally appropriate to improve the lives of people affected by mental health problems.

International alliances such as NEESAMA provide a valuable opportunity to bring together stakeholders across countries, regions and cultures to collaboratively address mental health challenges, leading to a more equitable and effective approach to mental healthcare globally.

## Data Availability

Data availability is not applicable to this article as no new data were created or analysed in this study.
